# Residue Analysis and Risk Exposure Assessment of Multiple Pesticides in Tomato and Strawberry and Their Products from Markets

**DOI:** 10.3390/foods12101936

**Published:** 2023-05-10

**Authors:** El-Sayed A. El-Sheikh, Dongyang Li, Ibrahim Hamed, Mohamed-Bassem Ashour, Bruce D. Hammock

**Affiliations:** 1 Plant Protection Department, Faculty of Agriculture, Zagazig University, Zagazig 44511, Al-Sharkia, Egypt; 2 Laboratory of Agricultural Information Intelligent Sensing, College of Biosystems Engineering and Food Science, Zhejiang University, Hangzhou 310058, China; 3 Department of Entomology and Nematology, and UCD Comprehensive Cancer Center, University of California, Davis, CA 95616, USA

**Keywords:** multi-residues, tomato, strawberry, sauce, jam, juice, dietary risks

## Abstract

Pesticides are used on fruit and vegetable crops to obtain greater yield and quality. Residues can be detected in these crops or their products if applied pesticides do not degrade naturally. Therefore, this study aimed to estimate pesticide residues in some strawberry and tomato-based products available in the market for human consumption and associated dietary risks. Contamination with 3–15 pesticides in the tested samples was found. The total number of pesticides detected in the tested samples was 20, belonging to the group of insecticides (84%) and fungicides (16%). Pesticides of cypermethrin, thiamethoxam, chlorpyrifos, and lambda-cyhalothrin appeared at 100% in a number of samples, where the most detected was cypermethrin followed by thiamethoxam. The average values of pesticide residues detected in the tested samples ranged from 0.006 to 0.568 mg kg^−1^, where it was found that cypermethrin had the highest residue value and appeared in strawberry jam obtained from the market. The recovery rate of pesticides from fortified samples with pyrethroids ranged from 47.5% (fenvalerate) to 127% (lambda-cyhalothrin). Home processing of fortified tomato and strawberry samples had a significant effect on reducing residues in tomato sauce and strawberry jam, where the reduction reached 100%. The results of acute and chronic risk assessment showed that their values were much lower than 100%, indicating minimal risk of dietary intake.

## 1. Introduction

Adequate agricultural production of food and fiber is more important than ever to feed a growing population and contribute to the overall development of many countries, especially developing ones. They depend on agriculture and should adopt more efficient and sustainable climate friendly production methods to ensure high crop yield [1]. Insect pests compete with humans for feeding and some of them can pose a major threat to an entire country or even a region as a fifth of the world’s total crop production could be destroyed annually by herbivorous insects [2]. It was reported by Kolani et al. [3] that around 45% of the crop production worldwide is lost annually due to pests and diseases.

Pesticides are used in agriculture in order to ensure high crop yields through controlling insect pests, plant pathogens, and weeds. They have been widely used throughout the world since the middle of the last century for their various benefits [4]. Since the banning of organochlorides, organophosphates (OPs), carbamates, and synthetic pyrethroids have become the most widely used classes of insecticides due to their high activity and relatively low persistence [5]. The toxicity of pyrethroids is considered high to insects due to the more sensitive sodium channels in the insect nervous system compared to mammals and birds [6]. At the same time, pyrethroids are considered less toxic than OPs and carbamates but are well known to affect human health in terms of their adverse effects on the immune system, fertility, liver and kidney enzyme activity, and the cardiovascular system. Accordingly, they induce nephro- and hepato-toxicity, inflammation, and influence the activity of antioxidant enzymes in mammal tissues [7]. While the current use of OPs and carbamates is decreasing, new pesticide groups are appearing on agriculture markets, including neonicotinoids, spinosyns, avermectins, diamides, and others. Diamides are a highly effective class of insecticide extensively used for the control of lepidopteran pests of tomatoes and strawberries in Egypt and many other countries due to their broad spectrum with high efficacy, as they are potent agonists of the insect ryanodine receptor (RyR) [8].

Fruits and vegetables are infected with many diseases and insect pests that need to be controlled as they are essential foodstuffs for different communities [9]. Often vegetables and fruits are eaten fresh without peeling or processing due to their high nutritional value and content of antioxidants, vitamins, minerals, and fiber [10,11,12]. Fruits and vegetables are also processed into ready-made food products sold in the markets, such as the production of fruit and vegetable juices, jams, sauces, ketchup, etc., which is considered one of the fastest growing industries for their important role in global food consumption [13].

Due to the long-term application of different pesticide groups pest and disease control on fruits, vegetables, and other field crops, soil, ground water, and reservoirs in many areas are now heavily contaminated. It has been shown that only 0.1% of applied chemical pesticides are effectively used for preventing, controlling, and mitigating pests, while the rest remains in the soil [14] causing toxicity to the biota and thereby entering into the food chain. Therefore, the contamination of fruits, vegetables, and other field crops possess a serious health risk to the public [15,16,17]. The impact of pesticide contamination on food has been well studied in North America, Japan, and many parts of Europe. In contrast, there is very little data on the levels of pesticide residues in developing countries, especially for those ready-made products available for direct use in markets. Food monitoring studies in Egypt were mainly limited to the analyses of organochlorides, organophosphates, and carbamate insecticides, which are well known to have adverse effects on human health and which are declining in use. Therefore, to protect consumers’ health, rapid and reliable identification and quantification of pesticides in raw and processed foods are critical to regulators, producers, and consumers [9].

However, with the increased use of pesticides, there will be an increased risk of occupational exposure for workers and non-occupational exposure for consumers of the produce. Repeated use may cause accumulation of pesticide residues in soil over time resulting in food contamination [18]. The health risks of pesticides are assessed on the basis of the total amount of pesticide residues detected in the samples analyzed based on the standard food intake rate of the groups being studied. Residues above certain limits in raw or processed food products have been shown to pose potential risks to the general population. Possible health risks were evaluated in samples of vegetables and fruits containing pesticide residues, which showed that consumers are exposed to health risks if the values of the acute reference dose (%ARfD) or tolerable daily intake (%ADI) are more than 100% [9].

Research and monitoring of these pesticides is a relatively new subject, and a knowledge gap still remains. Therefore, the aim of this study was to estimate pesticide residues in raw tomato and strawberry samples and their ready-made products available for direct consumption in local markets in Egypt. The effect of processing fortified raw tomato and strawberry samples with pyrethroid insecticides was determined. The dietary risk assessment of the detected pesticides in samples was also investigated.

## 2. Materials and Methods

### 2.1. Reagents and Materials

Seven analytical standard pyrethroid insecticides (bifenthrin, cypermethrin, deltamethrin, esfenvalerate, permethrin, lambda-cyhalothrin, and fenvalerate) with 99.1, 99.0, 99.9, 98.5, 91.8, 98.0, and 97.0% purity, respectively (Table S1 Supplementary Material) were purchased from Sigma-Aldrich (Steinheim, Germany). Acetonitrile, methanol, formic acid, and acetone were chromatographic grade and purchased from Merck (Darmstadt, Germany). Other chemicals of magnesium sulphate (MgSO4: 98% purity), sodium acetate (NaAc; 99% purity), primary secondary amine (PSA: 99% purity), sodium chloride (NaCl: 99% purity), and ammonium formate (98% purity) were purchased from Sigma-Aldrich (Steinheim, Germany).

### 2.2. Samples Collection

Fresh tomato and strawberry samples (25 samples each; 1 kg for each sample) were collected from local markets in Zagazig, SH, Egypt. Commercial products of strawberry and tomato (strawberry yogurt, strawberry juice, strawberry jam, dried strawberry, tomato sauce, tomato ketchup, tomato juice, and dried tomato) (3 samples of a complete package from each type) were obtained from different local markets in Zagazig. The product samples were stored at 4 °C, while the fruit samples were cut into small pieces and stored at −20 °C until use within one week.

### 2.3. Spiked Samples of Tomato and Strawberry with Pesticides

Samples of tomato and strawberry from markets (3 samples for each with 1 kg of each sample) were separately homogenized by using a laboratory blender (Model 8010S warring blinder, Chula Vista, CA, USA) for two minutes. The homogenized samples were spiked with seven insecticides of the pyrethroid group (Table S1 Supplementary Material). The final concentration after mixing with either tomato or strawberry was 2 mg kg^−1^ of each insecticide. Fortified samples (in triplicates) were left for 2 h under laboratory conditions before either extraction or processing into sauce (from fortified tomato samples) or jam (from fortified strawberry samples).

### 2.4. Spiked Samples of Products from Market with Pesticides

Tomato and strawberry-based products from markets (strawberry yogurt, strawberry jam, tomato sauce, and tomato ketchup) were spiked with four pyrethroid insecticides of deltamethrin, lambda-cyhalothrin, permethrin, and fenvalerate in order to determine the recovery percentages. Three concentrations prepared in acetone from each insecticide were mixed with tomato sauce, tomato ketchup, strawberry yogurt, and strawberry jam to give final concentrations of 0.01, 0.1, and 1 mg kg^−1^. Each sample (n = 3) was mixed with each concentration of the above four insecticides and left under laboratory conditions for two hours before extraction and clean up.

### 2.5. Home Processing of Fortified Tomato and Strawberry

Tomato samples: 300 g of each fortified tomato sample (final concentration of each insecticide 2 mg kg^−1^) (n = 3) with seven pyrethroid insecticides was boiled for 1 h until the water content completely evaporated. Before boiling, 5 g of NaCl was added and the final weight after processing fortified tomato samples into tomato sauce was around 200 g.

Strawberry samples: 300 g of each fortified strawberry sample (final concentration of each insecticide 2 mg kg^−1^) (n = 3) of seven pyrethroid insecticides was mixed with 150 g sugar (commercial sugar from local markets). The mixture was boiled for 1 h until the water content completely evaporated. The final weight of fortified strawberry sample after processing was around 250 g.

### 2.6. Sample Preparation

For the first extraction step, 10 g of the homogenized (tomato or strawberry) or market products (tomato sauce, tomato ketchup, strawberry yogurt, or strawberry jam) or home processed samples (tomato sauce or strawberry jam) of each sample was weighed in a 50 mL conical tube and vortexed with 10 mL of acetonitrile for 1 min. For the second extraction step, salts of magnesium sulphate (MgSO_4_; 4 g), sodium chloride (NaCl; 1 g), trisodium citrate dehydrate (1 g), and disodium hydrogen citrate sesquihydrate (0.5 g) were added to each tube, vortexed for 2 min, and centrifuged at 4000 rpm for 10 min [9]. Clean up by dispersive solid phase extraction (dSPE) was performed by transferring 4 mL of the resulting supernatant of each sample extraction into a 15 mL conical tube containing MgSO_4_ (300 mg) and PSA (50 mg) and centrifuged at 4000 rpm for 5 min. Following the clean-up process, the supernatant was decanted into clean tubes and acidified with formic acid to improve the storage stability of base-sensitive pesticides and then employed for LC- and GC-MS/MS analysis.

### 2.7. LC-MS/MS Analysis

Chromatographic analysis of pesticide residues with LC-MS/MS was determined using an Exion HPLC system (SCIEX) with a 6500+ QTRAP triple quadrupole mass spectrometer (AB SCIEX). The system operated in positive multiple reaction monitoring (MRM) modes for quantification and an electrospray ionization (ESI) source. A Zorbax XDB C18 column with 150 mm in length, 4.5 mm of inner diameter, and 5 μm of particle size (Agilent Technologies, Santa Clara, CA, USA) was used to perform the chromatography. The constant column temperature and flow rate were 40 °C throughout the analysis and 400 µL min^−1^, respectively, with an injection volume of 5 μL. Ammonium format (10 mM; pH 4.0) solution in water (90/10; *v*/*v*) (phase A) and methanol (phase B) were used as the mobile phases with gradient elution program as follows: 100% phase (A); 5% phase (A); 5% phase (A); 100% phase (A); 100% phase (A) after 0 min, 13.0 min, 21.0 min, 28.0 min, and 32.0 min, respectively. Analyst software Version 1.8.1 (Applied Biosystems) was used for data acquisition, processing for analyte confirmation, and quantitative analysis. The analytes were detected in the positive ionization mode over the m/z range from 50 to 1100, with ESI using MRM with MS/MS acquisition mode. Ion spray voltage, ion source temperature, and curtain gas were set as 5500 V, 400 °C, and 20 psi, respectively, as main source parameters. The following parameters derived from the flow rate were used: capillary voltage, 4000 V; fragmentor voltage, 190 V; drying gas, 9 L/min; drying gas temperature, 325 °C. Collision gas medium, nebulizer gas, and auxiliary gas were all set at 45 psi.

### 2.8. GC-MS/MS Analysis

Gas chromatography (GC, 7890A; Agilent, Palo Alto, CA, USA) coupled with a triple-quadrupole tandem mass spectrometer (MS/MS) (7010B) was used for pesticide residue analyses. Capillary column 30 m × 0.25 mm × 0.25 μm (Agilent J & W HP-5MS; Agilent Technologies, Santa Clara, CA, USA) with electron impact (EI) ionization source and helium (99.9% purity with a constant flow rate of 1 mL min^−1^) as a carrier gas were used for chromatographic separation. High-purity nitrogen was used as the collision cell gas with a flow rate of 1.5 mL min^−1^, and the quench gas was helium at 4 mL min^−1^. Sample volumes of 1.0 μL were injected in split/split less injection mode and a silica liner with a diameter of 2 mm was used. The initial temperature program was set to 40 °C for 2 min and then increased to 220 °C at a rate of 30 °C min^−1^. The oven temperature was then increased to 260 °C at a rate of 5 °C min^−1^ and then finally increased to 280 °C at 20 °C min^−1^ and held for 15 min. A fixed temperature of 250 °C was set for the split/splitless injector. The ion energy for electron impact was kept at 70 eV. MS1 and MS2 quadrupoles’ temperature was set at 150 °C, while the interface, manifold, and trap temperatures were set at 270, 50, and 210 °C, respectively. MRM transitions mode was used for quantitative and qualitative analysis of the compounds based on the most intensive precursor ion-product.

### 2.9. Dietary Risk Assessment

The acute and chronic risks of ingestion were assessed based on the weight of the group that mostly consumes the juices and yogurt, which was represented by the group of students (youth). The average body weight (b.wt) was estimated at 45 kg for this group and the lowest and highest rates of consumption were listed in Table S2 (Supplementary Materials).

The acute dietary intake was represented by the acute reference dose percentage (%ARfD) used to calculate the acute risk [9,19] in strawberry yogurt, strawberry juice, and tomato juice samples. Acceptable or not acceptable risks are based on the calculated %ARfD. The acute risk is considered acceptable when %ARfD is <100% and considered not acceptable when %ARfD is ≥100%. Accordingly, the lower risk is associated with the smaller %ARfD values, which was calculated according to Formulas (1) and (2):

(1) ESTI=(HFC×HRC)/b.wt.                       

(2) %ARfD=(ESTI×100)/ARfD               

where ESTI (Table S3 Supplementary Materials) is the estimated short-term intake (mg kg^−1^ day), HFC is the highest amount of food consumption by youth individuals in a day (kg), and HRC is the highest residual concentration detected for a pesticide (mg kg^−1^).

The risk associated with chronic dietary intake was calculated by the acceptable daily intake percentage (%ADI) [9,19] for each pesticide residue in strawberry yogurt, strawberry juice, and tomato juice samples. %ADI was calculated by Equations (3) and (4):

(3) NEDI=(AFC×APR)/b.wt.                   

(4) %ADI=(NEDI×100)/ADI                        

where NEDI (Table S3 supplementary materials) is the national estimated daily intake (mg kg^−1^ day), AFC is the average daily food consumption (kg), APR is average pesticide residue (mg kg^−1^), and ADI is the acceptable daily intake (mg kg^−1^ day). The chronic risk was considered acceptable or not acceptable when the %ADI < 100% or ≥100%, respectively. Therefore, the lower %ADI value represents a lower chronic risk [9].

The values of ADI and ARfD were obtained from the European Pesticide Database of the European Commission [20].

### 2.10. Statistical Analysis

The recovery data were analyzed using Statistical Package for the Social Sciences (SPSS, version 18.0) program with Duncan’s Multiple Range Test with a *p* < 0.05 significance value to determine statistical differences of recoveries among different concentrations within each product type.

## 3. Results

### 3.1. Residue Analysis of Pesticides in Tomato, Strawberry, and Their Product

**Figure 1:**
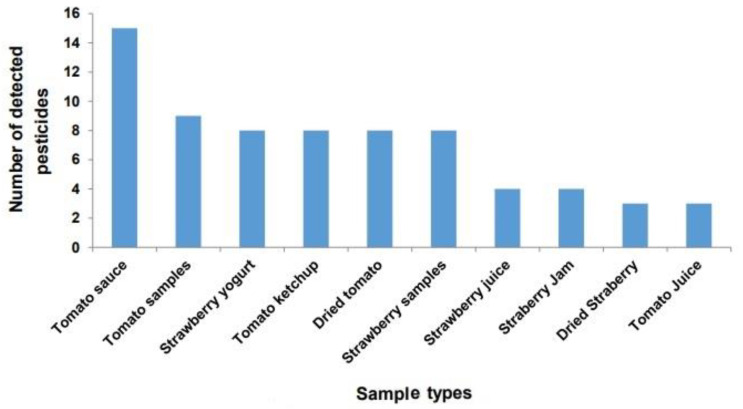
The total number of the detected pesticides (insecticides, fungicides, and herbicides) in each sample type.

The results presented in Figure 1 show the total number of pesticides that were detected in the tested samples by chromatographic analysis. From the results of the analysis, it was found that samples of tomato sauce contain the highest number of pesticides (15 pesticides). Tomato samples were contaminated with nine pesticides, while each of strawberry yogurt, tomato ketchup, dried tomatoes, and strawberry samples contained eight pesticides. Each of the strawberry juice and strawberry jam samples contained four pesticides, while each of dried strawberry and strawberry juice samples contained three pesticides.

The results presented in Table 1 show the analysis of the multi-pesticide residues in tomato and strawberry samples and their products from the market. The highest average pesticide residues in the tested samples were recorded for cypermethrin (0.568 mg kg^−1^) in strawberry jam, lambda-cyhalothrin (0.351 mg kg^−1^) in tomato sauce, pyridalyl (0.348 mg kg^−1^) in tomato sauce, cypermethrin (0.335 mg kg^−1^) in dried tomatoes, and cypermethrin (0.250 mg kg^−1^) in tomato samples. The lowest average value of residues was 0.006 mg kg^−1^ for acetamiprid, carbendazim, permethrin, difenoconazole, and chlorfenapyr, which were detected in the tomato sauce, tomato ketchup, dried tomato, tomato sample, and strawberry sample, respectively.

The percentage of pesticide detection in the tested samples of tomatoes, strawberries, and their products from the market shows that some pesticides were detected in 100% of the samples. It was found that cypermethrin was the most detected pesticide by 100% of the tested samples, as it was detected in strawberry yogurt, strawberry juice, strawberry jam, tomato sauce, tomato ketchup, tomato samples, and strawberry samples. Also, thiamethoxam was detected in 100% of strawberry jam, tomato sauce, and tomato ketchup samples, while chlorpyrifos and lambda-cyhalothrin were detected in 100% of tomato samples (Figure S1 Supplementary Materials).

**Figure 2:**
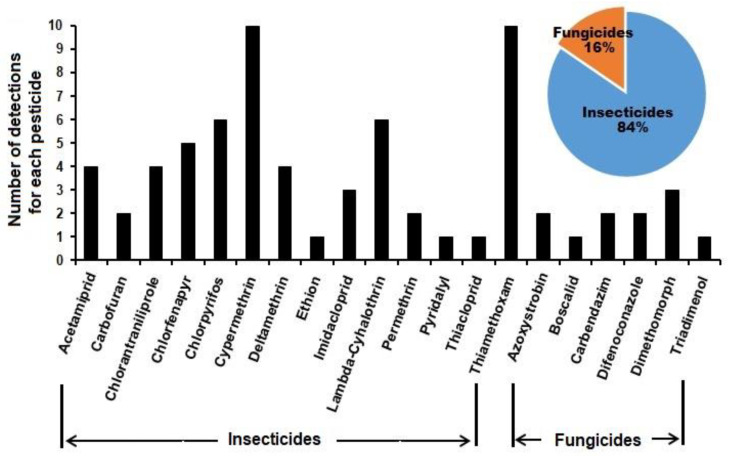
The number of detections for each pesticide classified according to groups (insecticides or fungicides), and the percentages of detection in all analysed tomato and strawberry samples and their products from the market.

The number of times each pesticide was detected in the tested samples ranged from only once (triadimenol, boscalid, thiacloprid, pyridalyl, and ethion) to ten times (cypermethrin and thiamethoxam). It was found that all the detected pesticides (20 pesticides) belonged to two groups: the insecticides (84%) and the fungicides (16%) (Figure 2).

### 3.2. Recovery of Pyrethroids from Tomato and Strawberry Based-Products

The recovery percentages from strawberry yogurt, strawberry jam, tomato sauce, and tomato ketchup were studied when fortified with four pyrethroids (deltamethrin, lambda-cyhalothrin, permethrin, and fenvalerate) (Table 2). It was found that the highest recovery rates were recorded as follows: 94.5 ± 4.9% of deltamethrin from strawberry jam for the fortified concentration of 0.01 mg kg^−1^; 118.0 ± 21.2% of lambda-cyhalothrin from tomato ketchup for the fortified concentration of 0.1 mg kg^−1^; and 127.9 ± 8.1% of lambda-cyhalothrin from tomato ketchup for the fortified concentration of 1.0 mg kg^−1^. Whereas the lowest recovery percentages were 47.5 ± 10.6%, 61.0 ± 2.8%, and 65.2 ± 4.0% of fenvalerate from fortified strawberry yogurt with concentrations of 0.01, 0.1, and 1.0 mg kg^−1^, respectively. In general, lambda-cyhalothrin was the most significant insecticide in the recovery at all fortified concentrations (Table 2).

The results presented in Table 3 show the effect of processing tomatoes into sauce and strawberries into jam on pesticide residues, especially those belonging to the group of pyrethroids when mixed at concentrations of 2 mg kg^−1^ with tomato or strawberry samples collected from the market. It was found that there was more than 96% reduction in the concentrations of all detected pesticide residues, except chlorpyrifos, whether in the samples of home-processed tomato sauce or strawberry jam. Among pyrethroid insecticides, a 100% reduction in lambda-cyhalothrin residues in both tomato sauce and strawberry jam due to the home processing of tomato and strawberry was found. The reduction percentages of chlorpyrifos were 42.6 and 51.8% in tomato sauce and strawberry jam, respectively (Table 3).

### 3.3. Dietary Risk Assessment

The highest percentage of acute risk was recorded with cypermethrin (62.15%) in strawberry yoghurt, lambda-cyhalothrin (29.39%) in strawberry yoghurt, and carbofuran (23.7%) in tomato juice. In the chronic risk, the highest percentages were recorded with carbofuran (23.7%) in tomato juice and lambda-cyhalothrin and cypermethrin in strawberry yogurt with 8.8 and 8.5%, respectively. All the obtained data of acute or chronic risks were much lower than 100%, which showed no significant risks when consuming these products (Table 4).

## 4. Discussion

About two million tons of pesticides are used annually worldwide [21]. Insecticides and fungicides represent approximately 48%, while herbicides account for approximately 50% [20]. Analysis of pesticide residues in commercial products manufactured from vegetables and fruits is necessary and indispensable at the present time. Due to the presence of pesticide residues in fresh vegetables and fruits, there is a high probability that these residues will remain in their commercial products. The system that assesses the risks posed by pesticides often depends on agricultural field products without considering the effects of different methods of processing on the presence or dissipation of residues in these final products [22].

Pesticide residues were detected in tomato and strawberry-based products from markets (Figure 1 and Figure 2; Table 1). The total number of detected pesticides was 20, belonging to both insecticides and fungicides with up to 15 pesticides detected in one sample type (tomato sauce), as reported in Table 1. Tomato is one of the most popular vegetable components in the diet in different regions of the world as it is consumed raw, cooked, or processed. Despite this, pesticides are applied extensively on tomato plants due to their exposure to many insect pests and fungal and bacterial diseases in order to control these pests to avoid significant losses in yield [23]. The result is detecting high numbers of pesticides in tomato available in farmer’s markets for human consumption [9]. A number of pesticides may appear in tomato-based products, as not all residues are eliminated during the process, which was confirmed by this study. Additionally, pesticide residues were detected in the products available in the market based on strawberries, where three and eight different pesticides were detected in dry strawberry and strawberry yogurt (Table 1), which indicates the transfer of pesticides to the processed products [24,25,26,27]. In agreement with our findings, pesticide residues were determined in concentrated apple juice samples by Hu et al. [28,29]. They detected 105 pesticide residues with a mean recovery of 70 to 100% in more than 90% of the detected pesticides. Different groups of pesticides were detected, including pyrethroids, which showed 15 pyrethroid residues in apple juice and developed LC- and GC-MS procedures for the determination of multiple pesticide residues in concentrated fruit and vegetable juices [30,31]. Pesticide residues exceeding MRLs ranged from 12.5 % (in tomato ketchup) to 50% (in both strawberry juice and strawberry jam). Eight pesticides exceeded MRLs in the tested samples, including raw tomato and strawberry (Table 1). This phenomenon was recorded by Hlihor et al. [32], who showed that chlorotalonil and bifenthrin exceeded the MRL in tomato after harvest.

The recovery experiments are useful in showing the efficiency and accuracy of both the extraction process and the estimation of pesticide residues. The closer the recovery percentage is to 100%, the more accurate the results of estimating the residues will be and the more accurate the detection of the amount of pesticide present in the tested samples will be. A recovery of more than 80% for pyrethroids in general and up to 128% for lambda-cyhalothrin was obtained when the samples were fortified with a concentration of 1.0 mg kg^−1^ (Table 2). The recovery % was studied for different food matrices and showed different recovery percentages depending on the type of sample [33,34,35]. Our findings are consistent with the results of Huang et al. [36], who obtained more than 80% recovery of pyrethroid insecticides from different samples of tea and suggested that the established method exhibits a satisfactory performance for the determination of pyrethroid residues in tea drinks. Additionally, Arisekar et al. [37] showed up to 94% recovery in seafood for methoxychlor.

Results of the current study showed that the home processing of tomato and strawberry into sauce and jam, respectively, significantly reduced the residue levels of pesticides, reaching 100% reduction with some types of pesticides. This reduction contributes to increasing food safety related to pesticide contamination. Different simple methods were used in treating food samples for decreasing residues. Washing, heating, and drying are among the simple methods for removing pesticide residues [9,35,38]. The washing technique using ozone microbubble treatment as an eco-friendly method was applied on pakchoi, celery, and cowpea vegetables and showed up to 94% removal of emamectin benzoate [39]. Bai et al. [38] reported that the steaming process reduced the residues of eighteen pesticides, including chlorantraniliprole, azoxystrobin, difenoconazole, and imidacloprid, by 32.0–75.3% through evaporation or thermal degradation. Li et al. [40] showed that washing and peeling can reduce the cyenopyrafen residues in strawberry (washing) and mandarin (peeling) samples by 46 and 100%, respectively. Meanwhile, they reported an increase in the amount of residues due to processing by converting strawberry into jam and mandarin into juice by 1.8 and 1.3 times, respectively, which is contrary to what we obtained in this study. This might be due to the differences in the chemical and physical properties of cyenopyrafen compared to other pesticides detected in our study. Moreover, in the same context of our findings, Naman et al. [41] showed that washing, peeling, and heat processing (boiling and blanching) were the most effective ways of pesticide residue dissipation. Wongmaneepratip et al. [33] investigated the effects of the different processing methods of thermal (grilling and boiling) and non-thermal (pickling and curing) treatments and freeze storage on the reduction of cypermethrin, deltamethrin, bifenthrin, and permethrin in mackerel fillets. They reported that curing and frozen storage processes were the most effective methods in reducing pyrethroid residues (>70% reduction) from fish fillets.

When manufacturing products from raw materials (as in strawberries and tomatoes), a decrease in the concentration of a pesticide usually occurs. While the number of substances detected in the products may increase and cause potential toxicity is humans [42]. Our research confirmed this phenomenon, as 15 pesticides were detected in tomato sauce, while nine pesticides were detected in raw tomato samples. This phenomenon is explained by the presence of a number of pesticides with residues less than the limit of detection (LOD). After concentrating the sample volume through processing (such as converting tomatoes into sauce or strawberries into jam), the residue values of these pesticides become higher than the LOD. Therefore, some processed samples may contain pesticide residues higher than their original raw samples, especially with heat-stable pesticides [9]. This finding is consistent with what was obtained by Sójka et al. [43], where the press cake resulting from the strawberry processing was found to contain more than eight pesticides, and the fruit itself contained only two on average. Some pesticides showed stability after processing, i.e., chlorpyrifos only showed a 43 and 52% reduction after the home processing of tomato and strawberry into sauce and jam, respectively, while lambda-cyhalothrin showed a 100% reduction. This finding was confirmed by the study of Naman et al. [41], who showed that the reduction in pesticide residues of mancozeb after processing tomato into sauce and apple into jam were 38 and 100%, respectively.

Results of dietary risk assessment showed that the acute (%ARfD) risk ranged from 0.01 to 62.15% and the chronic (%ADI) risk ranged from 0.01 to 23.7% when testing strawberry yogurt, strawberry juice, and tomato juice (Table 4). Both acute and chronic risk values were much less than 100%, reflecting minimum or no associated risk. In agreement with our results, Hlihor et al. [32] showed that the risks generated by the residues of pesticides detected in tomatoes were in acceptable values, except chlorothalonil, which may pose a threat to children’s health when sprayed with recommended or double recommended doses. When they considered the values of the cumulative hazard index, they found that risks were higher than 1, which indicates that the consumption of contaminated tomatoes may cause harmful non-carcinogenic health effects. Long-term dietary exposure to cyenopyrafen was found to be 73.7% when determined in strawberry, indicating that consuming it was unlikely to cause a public health concern [40]. Risk assessment of different samples (tomato, potato, pear, apricot, peach, and apple) before and after processing was evaluated by Naman et al. [41]. When comparing the maximum residue contribution between samples before and after processing, they found that residues were much less in the processed samples and that processed food samples are fit to be consumed.

## 5. Conclusions

The increase in food and fiber production is associated with the widespread use of different groups of pesticides to suppress pest populations on agricultural crops. As a result of the long-term use of pesticides, soil, groundwater, and the food chain are heavily contaminated. Therefore, the contamination of fruits, vegetables, and their products poses serious health risks to consumers. Since there is a lack of data regarding pesticide residues in processed food, this study aimed to monitor pesticide residues and investigate the associated dietary risks in strawberry and tomato-based products available in markets. The results of this study showed the presence of residues of multiple pesticides in strawberry yoghurt, strawberry juice, strawberry jam, tomato sauce, tomato juice, tomato ketchup, and dried strawberries and tomatoes, ranging from 3 to 15 pesticides in each sample. Additionally, 27% of the average pesticide residues in the tested samples were higher than the MRLs. When evaluating the risks of acute and chronic consumption of the tomato and strawberry-based products (strawberry yogurt, strawberry juice, and tomato juice), it was found that there were no potential acute or chronic risks. Therefore, rapid and reliable identification and quantification of pesticides in raw and processed foods is critical to regulators and producers to ensure the safety and security of food and to preserve the health of consumers.

## Figures and Tables

**Table 1:** Multi-residue analysis (residue range, average, RSD, LOD, and LOQ) of pesticides in strawberry and tomato and their products.

Samples	Pesticides	Residue (mg kg^−1^)			LOD (mg kg^−1^)	LOQ (mg kg^−1^)	MRL * (mg kg^−1^)
		Range	Average	RSD			
Strawberry yogurt	Acetamiprid	0.019–0.019	0.019	0.000	0.0001	0.0010	0.050
	Chlorantraniliprole	0.011–0.013	0.012	0.001	0.0001	0.0010	0.050
	Chlorpyrifos	0.006–0.022	0.013	0.008	0.0016	0.0032	0.010
	Cypermethrin	0.015–0.368	0.118	0.126	0.0040	0.0230	0.100
	Imidacloprid	0.014–0.014	0.014	0.000	0.0001	0.0050	0.050
	Lambda-Cyhalothrin	0.024–0.174	0.106	0.090	0.0001	0.0010	0.010
	Thiacloprid	0.025–0.124	0.075	0.070	0.0001	0.0010	50.000
	Thiamethoxam	0.008–0.046	0.020	0.018	0.0017	0.0050	0.050
Strawberry juice	Chlorantraniliprole	0.011–0.126	0.069	0.081	0.0001	0.0010	0.050
	Cypermethrin	0.032–0.096	0.066	0.032	0.0040	0.0230	0.100
	Lambda-Cyhalothrin	0.011–0.011	0.011	0.000	0.0001	0.0010	0.010
	Thiamethoxam	0.024–0.024	0.024	0.000	0.0017	0.0050	0.050
Strawberry jam	Acetamiprid	0.013–0.013	0.013	0.000	0.0001	0.0010	0.050
	Chlorantraniliprole	0.014–0.147	0.081	0.094	0.0001	0.0010	0.050
	Cypermethrin	0.018–2.739	0.568	1.073	0.0040	0.0230	0.100
	Thiamethoxam	0.010–0.083	0.030	0.027	0.0017	0.0050	0.050
Dried strawberry	Cypermethrin	0.215–0.215	0.215	0.000	0.0040	0.0230	0.100
	Deltamethrin	0.020–0.020	0.020	0.000	0.0018	0.0100	15.000
	Thiamethoxam	0.050–0.050	0.050	0.000	0.0017	0.0050	0.050
Tomato sauce	Acetamiprid	0.006–0.006	0.006	0.000	0.0001	0.0010	0.500
	Azoxystrobin	0.007–0.021	0.011	0.007	0.0013	0.0040	3.000
	Boscalid	0.015–0.015	0.015	0.000	0.0001	0.0010	3.000
	Carbendazim	0.008–0.009	0.009	0.001	0.0001	0.0010	0.300
	Chlorantraniliprole	0.006–0.035	0.021	0.021	0.0001	0.0010	0.600
	Chlorfenapyr	0.021–0.031	0.027	0.004	0.0025	0.0100	0.010
	Chlorpyrifos	0.006–0.030	0.012	0.010	0.0016	0.0032	0.010
	Cypermethrin	0.038–0.788	0.176	0.249	0.0040	0.0230	0.500
	Difenoconazole	0.007–0.012	0.009	0.003	0.0001	0.0050	2.000
	Dimethomorph	0.007–0.023	0.013	0.007	0.0010	0.0050	1.000
	Imidacloprid	0.006–0.035	0.016	0.010	0.0001	0.0050	0.300
	Lambda-Cyhalothrin	0.009–1.311	0.351	0.579	0.0001	0.0010	0.070
	Pyridalyl	0.348–0.348	0.348	0.000	0.0005	0.0010	1.500
	Thiamethoxam	0.010–0.017	0.013	0.002	0.0017	0.0050	0.200
	Triadimenol	0.011–0.011	0.011	0.000	0.0005	0.0010	0.300
Tomato ketchup	Carbendazim	0.006–0.006	0.006	0.000	0.0001	0.0010	0.300
	Chlorfenapyr	0.011–0.016	0.014	0.003	0.0025	0.0100	0.010
	Chlorpyrifos	0.006–0.013	0.010	0.004	0.0016	0.0032	0.010
	Cypermethrin	0.026–0.066	0.050	0.020	0.0040	0.0230	0.500
	Deltamethrin	0.070–0.070	0.070	0.000	0.0018	0.0100	0.070
	Dimethomorph	0.007–0.028	0.018	0.015	0.0010	0.0050	1.000
	Imidacloprid	0.008–0.027	0.018	0.013	0.0001	0.0050	0.300
	Thiamethoxam	0.013–0.128	0.047	0.055	0.0017	0.0050	0.200
Tomato juice	Carbofuran	0.008–0.008	0.008	0.000	0.0001	0.0005	0.002
	Cypermethrin	0.067–0.067	0.067	0.000	0.0040	0.0230	0.500
	Thiamethoxam	0.013–0.013	0.013	0.000	0.0017	0.0050	0.200
Dried tomato	Acetamiprid	0.007–0.007	0.007	0.000	0.0001	0.0010	0.050
	Chlorfenapyr	0.015–0.015	0.015	0.000	0.0025	0.0100	0.010
	Chlorpyrifos	0.020–0.020	0.020	0.000	0.0016	0.0032	0.010
	Cypermethrin	0.335–0.335	0.335	0.000	0.0040	0.0230	0.500
	Dimethomorph	0.010–0.010	0.010	0.000	0.0010	0.0050	1.000
	Lambda-Cyhalothrin	0.033–0.033	0.033	0.000	0.0001	0.0010	0.070
	Permethrin	0.006–0.006	0.006	0.000	0.0001	0.0010	0.050
	Thiamethoxam	0.025–0.025	0.025	0.000	0.0017	0.0050	0.200
Tomato samples	Azoxystrobin	0.008–0.011	0.010	0.002	0.0013	0.0040	3.000
	Chlorfenapyr	0.017–0.022	0.020	0.004	0.0025	0.0100	0.010
	Chlorpyrifos	0.097–0.115	0.103	0.010	0.0016	0.0032	0.010
	Cypermethrin	0.015–0.681	0.250	0.374	0.0040	0.0230	0.500
	Deltamethrin	0.102–0.14	0.121	0.027	0.0018	0.0100	0.070
	Difenoconazole	0.006–0.006	0.006	0.000	0.0001	0.0050	2.000
	Lambda-Cyhalothrin	0.014–0.089	0.053	0.038	0.0001	0.0010	0.070
	Permethrin	0.075–0.075	0.075	0.000	0.0001	0.0010	0.050
	Thiamethoxam	0.010–0.013	0.012	0.002	0.0017	0.0050	0.200
Strawberry samples	Carbofuran	0.008–0.008	0.008	0.000	0.0001	0.0005	0.050
	Chlorfenapyr	0.006–0.006	0.006	0.000	0.0025	0.0100	0.050
	Chlorpyrifos	0.029–0.052	0.041	0.016	0.0016	0.0032	0.010
	Cypermethrin	0.059–0.228	0.147	0.085	0.0040	0.0230	0.100
	Deltamethrin	0.228–0.228	0.228	0.000	0.0018	0.0100	15.000
	Ethion	0.026–0.029	0.028	0.002	0.0037	0.0110	0.050
	Lambda-Cyhalothrin	0.008–0.045	0.026	0.009	0.0001	0.0010	0.010
	Thiamethoxam	0.011–0.043	0.027	0.023	0.0017	0.0050	0.050

* Maximum residue limit (MRL) for each pesticide was obtained from European commission pesticide residue database. MRLs of strawberry yogurt, strawberry juice, strawberry jam, dried strawberry, and strawberry samples are based on data registered for strawberry on European commission pesticide residue database. MRLs of tomato sauce, tomato ketchup, tomato juice, dried tomato, and tomato samples are based on data registered for tomato on European commission pesticide residue database.

**Table 2:** Percentages of recovery of pyrethroid insecticides from strawberry and tomato-based products.

Samples	Pesticides	Spiked Concentrations					
		Average *			RSD		
		0.01 (mg kg^−1^)	0.1 (mg kg^−1^)	1 (mg kg^−1^)	0.01 (mg kg^−1^)	0.1 (mg kg^−1^)	1 (mg kg^−1^)
Strawberry yogurt	Deltamethrin	65.0 ^b^	77.5 ^b^	94.1 ^b^	7.1	4.9	5.9
	Lambda-cyhalothrin	90.0 ^a^	105.0 ^a^	117.4 ^a^	14.1	1.4	10.5
	Permethrin	55.0 ^c^	75.5 ^b^	84.2 ^b^	7.1	2.1	7.1
	Fenvalerate	47.5 ^d^	61.0 ^c^	65.2 ^c^	10.6	2.8	4.0
Strawberry jam	Deltamethrin	94.5 ^a^	109.5 ^a^	106.7 ^a^	4.9	19.1	6.3
	Lambda-cyhalothrin	89.5 ^a^	90.0 ^a^	120.1 ^a^	13.4	1.4	23.7
	Permethrin	75.5 ^b^	87.0 ^ab^	97.7 ^ab^	6.4	1.4	4.6
	Fenvalerate	62.5 ^c^	77.5 ^b^	85.5 ^c^	3.5	3.5	6.5
Tomato sauce	Deltamethrin	76.1 ^b^	101.0 ^a^	113.7 ^b^	8.6	2.8	1.8
	Lambda-cyhalothrin	87.5 ^a^	110.0 ^a^	121.2 ^a^	3.5	15.6	7.3
	Permethrin	72.5 ^b^	88.0 ^b^	84.5 ^c^	3.5	5.7	2.8
	Fenvalerate	55.5 ^c^	81.9 ^b^	83.0 ^c^	6.4	4.2	4.5
Tomato ketchup	Deltamethrin	77.5 ^b^	82.5 ^b^	109.2 ^b^	3.5	3.5	1.5
	Lambda-cyhalothrin	89.0 ^a^	118.0 ^a^	127.9 ^a^	1.4	21.2	8.1
	Permethrin	67.5 ^b^	74.5 ^bc^	89.3 ^c^	10.6	10.6	1.1
	Fenvalerate	57.5 ^c^	71.5 ^c^	82.9 ^c^	4.9	3.5	9.5

* Average values (mg kg^−1^) in columns for each concentration and each product type carrying different superscript letters are statistically different at *p* < 0.05 when analyzed using SPSS 18 software.

**Table 3:** Effect of tomato and strawberry processing into sauce and jam on residues of pesticides/pyrethroids.

Pesticides	Market Sample	Spiked Sample	Processed Sample *	% of Reduction **
Tomato				
Azoxystrobin	0.010	0.011	ND ***	100.0
Bifenthrin	ND	2.315	0.013	99.4
Chlorfenpayer	0.020	0.020	ND	100.0
Chlorpyrifos	0.052	0.054	0.031	42.6
Cypermethrin	0.250	2.438	0.010	99.6
Deltamethrin	0.121	2.603	0.002	99.9
Difenoconazole	0.006	0.007	ND	100.0
Esfenvalerate	ND	1.981	0.009	99.5
Fenvalerate	ND	2.162	0.013	99.4
Lambda-cyhalothrin	0.053	2.591	ND	100.0
Permethrin	0.075	2.079	0.011	99.5
Thiamethoxam	0.012	0.011	ND	100.0
Strawberry				
Bifenthrin	ND	1.716	0.020	98.8
Carbofuran	0.008	0.009	ND	100.0
Chlorfenpayer	0.006	0.006	ND	100.0
Chlorpyrifos	0.168	0.166	0.080	51.8
Cypermethrin	0.147	1.828	0.020	98.9
Deltamethrin	0.228	2.414	0.021	99.1
Esfenvalerate	ND	2.105	0.022	99.0
Ethion	0.028	0.028	ND	100.0
Fenvalerate	ND	2.212	0.080	96.4
Lambda-cyhalothrin	0.051	2.080	ND	100.0
Permethrin	ND	2.040	0.019	99.1
Thiamethoxam	0.027	0.026	ND	100.0

* Tomato sauce is the processed sample of tomato, while strawberry jam is the processed sample of strawberry. ** Percentage (%) of reduction in residues of pesticides was calculated for processed and spiked samples. *** ND = no detection, which considered zero (0) when calculating % of reduction.

**Table 4:** Acute (%ARfD) and chronic (%ADI) risk assessment of dietary intake of strawberry yogurt, strawberry juice, and tomato juice contaminated with pesticides.

Samples	Pesticides	ARfD * (mg kg^−1^ bw)	%ARfD	ADI * (mg kg^−1^ bw.day)	%ADI
Strawberry yogurt	Chlorpyrifos	0.005	3.72	0.001	3.11
	Cypermethrin	0.005	62.15	0.005	8.51
	Lambda-Cyhalothrin	0.005	29.39	0.0025	8.80
	Chlorantraniliprole	1.56	0.01	1.56	0.01
	Thiamethoxam	0.5	0.08	0.26	0.02
	Thiacloprid	0.02	5.24	0.01	1.66
	Acetamiprid	0.025	0.64	0.025	0.17
	Imidacloprid	0.08	0.15	0.06	0.05
Strawberry juice	Cypermethrin	0.005	10.02	0.005	5.69
	Lambda-Cyhalothrin	0.005	1.15	0.0025	1.96
	Chlorantraniliprole	1.56	0.04	1.56	0.02
	Thiamethoxam	0.5	0.03	0.26	0.04
Tomato juice	Cypermethrin	0.005	5.96	0.005	5.96
	Thiamethoxam	0.5	0.01	0.26	0.02
	Carbofuran	0.00015	23.70	0.00015	23.70

* ARfD and ADI values were obtained from the European Commission database.

## Data Availability

All related data and methods are presented in this manuscript and Supplementary Materials. Additional inquiries should be addressed to the corresponding author.

## Supplementary Materials

The following supporting information can be downloaded at https://www.mdpi.com/article/10.3390/foods12101936/s1. Table S1: Characteristics of pyrethroid insecticides; Figure S1: The percentages of the presence of pesticides in tomato and strawberry and their products from supermarkets. Strawberry yogurt (A), strawberry juice (B), strawberry jam (C), dried strawberry (D), tomato sauce (E), tomato ketchup (F), tomato juice (G), dried tomato (H), tomato samples (I), and strawberry samples (J); Table S2: Lower and higher daily consumption rate of strawberry yogurt, strawberry juice, and tomato juice; and Table S3: ESTI and NEDI for pesticide residues detected in strawberry yogurt, strawberry juice, and tomato juice.
