# Supplementary material for: Residue Analysis and Risk Exposure Assessment of Multiple Pesticides in Tomato and Strawberry and Their Products from Markets
Source: Foods. 2023 May 10;12(10):1936. doi: 10.3390/foods12101936 (PMC10217756; doi:10.3390/foods12101936)
Supplement: Supplementary file 1 [file foods-12-01936-s001.zip › foods-2331369-supplementary.pdf]

Supplementary materials:

Residue Analysis and Risk Exposure Assessment of Multiple Pesticides in Tomato and Strawberry and Their Products from Markets

El-Sayed A. El-Sheikh [1,*], Dongyang Li [2,3], Ibrahim Hamed [1], Mohamed-Bassem Ashour [1] and Bruce D. Hammock [3,*]

[1] Plant Protection Department, Faculty of Agriculture, Zagazig University, Zagazig 44511, Al-Sharkia, Egypt
[2] Laboratory of Agricultural Information Intelligent Sensing, College of Biosystems Engineering and Food Science, Zhejiang University, Hangzhou 310058, China
[3] Department of Entomology and Nematology, and UCD Comprehensive Cancer Center, University of California, Davis, CA 95616, USA
* Correspondence: eaelsheikh@zu.edu.eg (E.-S.A.E.-S.); bdhammock@ucdavis.edu (B.D.H.)

Table S1. Characteristics of pyrethroid insecticides.

No	Active substance	CAS-No	Structure	Molecular weight ($g\ mol^{-1}$)	Physical state	Melting point/range (°C)	Chemical stability
**1.**	Bifenthrin	82657-04-3	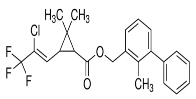	422.87	powder	66-71	Stable under RT *
**2.**	cypermethrin	52315-07-8	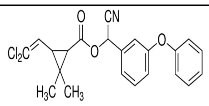	416.30	semi-solid	65-74	Stable under RT
**3.**	deltamethrin	52918-63-5	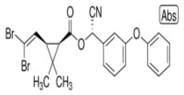	505.20	crystals	98-103	Stable under RT
**4.**	esfenvalerate	66230-04-4	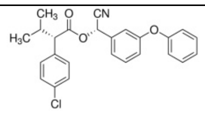	419.90	powder	58-64	Stable under RT
**5.**	permethrin	52645-53-1	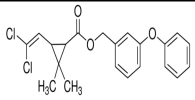	391.29	solid	34-35	Stable under RT
**6.**	lambda-cyhalothrin	91465-08-6	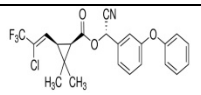	449.85	solid	49.2	Stable under RT
**7.**	fenvalerate	51630-58-1	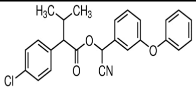	419.9	solid	59-63	Stable under RT

* RT= room temperature.

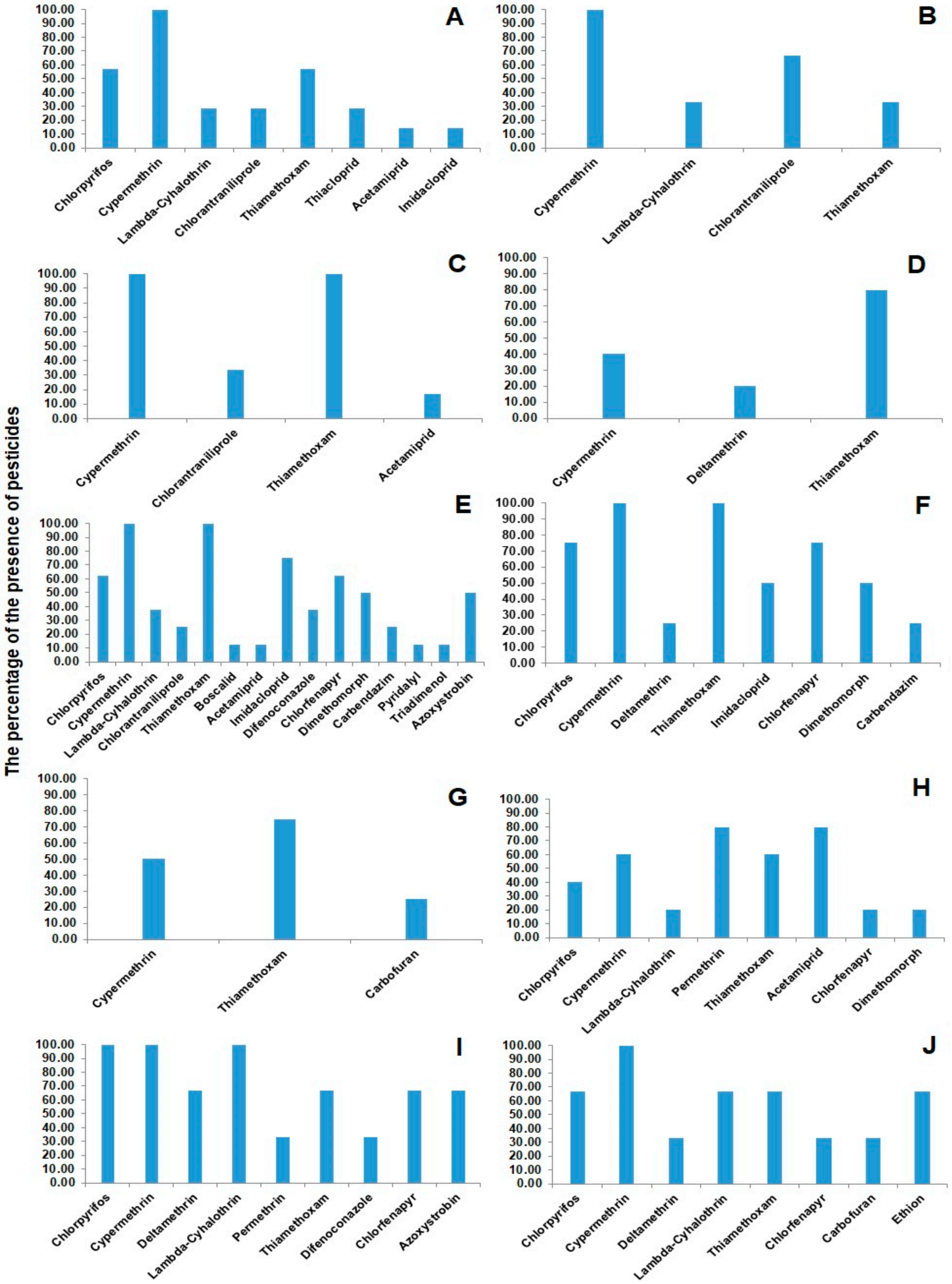

Figure S1. The percentages of the presence of pesticides in tomato and strawberry and their products from supermarkets. Strawberry yogurt (A), strawberry juice (B), strawberry jam (C), dried strawberry (D), tomato sauce (E), tomato ketchup (F), tomato juice (G), dried tomato (H), tomato samples (I), and strawberry samples (J).

Table S2: Lower and higher daily consumption rate of strawberry yogurt, strawberry juice, and tomato juice.

Samples	Consumption (Kg)	
	Lower	Higher
Strawberry yogurt	0.100	0.38
Strawberry juice	0.200	0.235
Tomato Juice	0.200	0.200

Table S3: ESTI and NEDI for pesticide residues detected in strawberry yogurt, strawberry juice, and tomato juice.

Samples	Pesticides	Residue (mg Kg^{-1})		ESTI	NEDI
		max	Average	(mg kg^{-1} day)	(mg kg^{-1} day)
Strawberry yogurt	Chlorpyrifos	0.022	0.014	0.000186	0.000031
	Cypermethrin	0.368	0.1915	0.003108	0.000426
	Lambda-Cyhalothrin	0.174	0.099	0.001469	0.000220
	Chlorantraniliprole	0.013	0.012	0.000110	0.000027
	Thiamethoxam	0.046	0.027	0.000388	0.000060
	Thiacloprid	0.124	0.0745	0.001047	0.000166
	Acetamiprid	0.019	0.019	0.000160	0.000042
	Imidacloprid	0.014	0.014	0.000118	0.000031
Strawberry juice	Cypermethrin	0.096	0.064	0.000501	0.000284
	Lambda-Cyhalothrin	0.011	0.011	0.000057	0.000049
	Chlorantraniliprole	0.126	0.0685	0.000658	0.000304
	Thiamethoxam	0.024	0.024	0.000125	0.000107
Tomato Juice	Cypermethrin	0.067	0.067	0.000298	0.000298
	Thiamethoxam	0.013	0.013	0.000058	0.000058
	Carbofuran	0.008	0.008	0.000036	0.000036
